# Impact of Web-Based Self-Scheduling on Finalization of Well-Child Appointments in a Primary Care Setting: Retrospective Comparison Study

**DOI:** 10.2196/23450

**Published:** 2021-03-18

**Authors:** Frederick North, Elissa M Nelson, Rebecca J Majerus, Rebecca J Buss, Matthew C Thompson, Brian A Crum

**Affiliations:** 1 Division of Community Internal Medicine Department of Medicine Mayo Clinic Rochester, MN United States; 2 Enterprise Office of Access Management Mayo Clinic Rochester, MN United States; 3 Department of Neurology Mayo Clinic Rochester, MN United States

**Keywords:** electronic health record, schedules, patient appointment, preventive health service, office visit, outpatient care, software tool, computer software application, mobile applications, child health, pediatric, preventive care, self

## Abstract

**Background:**

Web-booking of flights, hotels, and sports events has become commonplace in the travel and entertainment industry, but self-scheduling of health care appointments on the web is not yet widely used. An electronic health record that integrates appointment scheduling and patient web-based access to medical records creates an opportunity for patient self-scheduling. The Mayo Clinic developed and implemented a feature in its Patient Online Services (POS) web and mobile platform that allows software-managed self-scheduling of well-child visits.

**Objective:**

This study aims to examine the use of a new self-scheduling appointment feature within POS in both web and mobile formats and determine the use characteristics, outcomes, and efficiency of self-scheduling compared with staff scheduling.

**Methods:**

Within a primary care setting, we collected 13 months of all appointment activity for the well-child visit for children aged 2-12 years. As these specific appointment types are for minors, self-scheduling is performed by parents or other proxies. We compared the appointment actions of scheduling and cancelling for both self-scheduled and staff-scheduled appointments. The frequency in which patients were using self-scheduling outside of normal business hours was quantified, and we compared no-show outcomes of finalized appointments.

**Results:**

Of the 1099 patients who performed any self-scheduling actions, 73.1% (803/1099) exclusively used self-scheduling and self-cancelling software. For those with access to self-scheduling (patients registered with the Mayo Clinic POS), 4.92% (1201/24,417) of all well-child appointment-scheduling actions were self-scheduled. Staff scheduling required more than a single appointment step (eg, schedule, cancel, reschedule) in 28.32% (3729/13,168) compared with only 6.93% (53/765) of self-scheduled appointments (*P*<.001). Self-scheduling appointment actions took place outside of regular business hours 29.5% (354/1201) of the time. No-shows accounted for 3.07% (28/912) of the self-scheduled finalized appointments compared with 4.12% (693/16,828) of staff-scheduled appointments, which is a nonsignificant difference (*P*=.12). Staff-scheduled finalized appointments (that allowed for scheduling appointments for more than 12 weeks in the future) revealed a potential demand of 11.15% (1876/16,828) for appointments with longer lead times.

**Conclusions:**

Self-scheduling can generate a significant number of finalized appointments, decreasing the need for staff scheduler time. We found that 29.5% (354/1201) of the self-scheduling activity took place outside of the usual staff scheduler hours, adding convenience value to the scheduling process. For exclusive self-schedulers, 93.1% (712/765) finalized the appointment in a single step. The no-show rates were not adversely affected by the self-scheduling.

## Introduction

The travel and entertainment industries have provided web booking of flights, hotel rooms, and sports and entertainment events for many years, whereas web-based scheduling of medical appointments is not widely available. Gupta and Denton [1] summarized many of the unique challenges in health care that make scheduling rules for medical appointments complex and difficult to code into software. Ahmadi-Javid et al [2] reviewed much of the extant literature on outpatient appointment scheduling and decision making involved in the appointment-scheduling processes. In addition to the complex rules needed for scheduling, there are patient barriers to web-based appointment scheduling. A survey in Australia showed that 89% of primary care patients with access to a web-based appointment-scheduling system were reluctant to adopt it [3]. Although all patients had access to the system, only 11% used the web-based appointment service at least once, and 74% were not inclined to use the web-based appointment service in the near future. In interviews, some of the patients preferred phone call appointments that they perceived “provided them with more opportunities to discuss the options for more complex situations than the online self-service” [3]. Others cited low computer literacy and lack of access to the internet at home [3].

Despite these barriers, independent vendors are filling some demand for medical appointments on the web. ZocDoc (TM), for example, has been offering web-based appointment scheduling for health care practices [4]. Kurtzman et al [5] assessed appointments from 4150 physicians available in 20 cities where ZocDoc was available and found a “substantial number of appointments available for patients on ZocDoc,” with the conclusion that “ZocDoc is a promising method for obtaining reliable primary care appointments in the cities evaluated” [5]. Internationally, similar web-based platforms for self-scheduling health care appointments exist, such as Lybrate in India [6]. Zhao et al [7] reviewed the literature on web-based appointment systems and found support for associations between web-based appointment systems and improved no-show rates, decreased waiting time, improved patient satisfaction, and decreased staff labor.

Mayo Clinic implemented a web- and mobile-based self-scheduling option for a well-child visit in 2019. We examined the patient uptake and outcomes of the self-scheduling option to see if there were differences in use and appointment outcomes between self-scheduling and the use of Mayo staff appointment schedulers.

## Methods

### Setting

This study was conducted in the Mayo Clinic Health System in the Rochester, Minnesota, and Northwest Wisconsin regions for clinic well-child visits scheduled for the 13-month time interval from February 1, 2019, to February 28, 2020. Providers eligible to have well-child visits on their schedules were physicians, physician assistants, and nurse practitioners in family medicine and community pediatrics departments.

The Mayo Clinic uses Epic as its electronic health record (EHR) system. The Mayo Clinic has a patient portal, named Patient Online Services (POS), that patients can access via a mobile app or on the web. With the Mayo Clinic POS, patients can communicate with providers via secure messages, review their medical records, and view future appointment details. Patients of the Mayo Clinic have been increasingly engaged with POS, and portal registration has increased from 33% in 2013 to 62% in 2018 [8]. Although POS has been available for many years, self-scheduling of office visits through the POS has been made available only recently.

The self-scheduling process required POS, so portal registration was a prerequisite for self-scheduling. In this study, we use self-scheduling as a generic term for scheduling via software, without assistance from a staff scheduler. Owing to age limitations on the well-child appointment, self-scheduling and self-cancellations were accomplished by patient proxies such as parents or other adults who had portal access to the child’s EHR.

Well-child appointment scheduling with a staff scheduler was generally limited to Monday through Friday, from 7 AM to 5 PM. For self-scheduling, there was 24/7 access to a web-based self-scheduling process and mobile self-scheduling app, except for rare occasions of a software outage.

### Well-Child Appointment Type

The well-child examination is a periodic exam recommended by the American Academy of Pediatrics [9]. The Mayo-implemented self-scheduling feature is limited to exams for ages 2 to 12 years to decrease the complexity of rules associated with scheduling.

The well-child visit type is a good visit type for self-scheduling in primary care practice. By definition, the appointment is for a healthy child, so no symptoms require an urgent visit. Many appointments in primary care are symptom-based and can require some symptom assessment to determine the urgency of the visit. Self-scheduling symptom-based visits are a larger informatics challenge because of the patient safety issue around the urgency of visits that is not present in the well-child visit type.

The well-child visit is also a visit type that allows the provider some autonomy to schedule these visits in blocks or spread out, earlier or later in the day, or for a specific day of the week. The same visit type was used for self-scheduling as was used for staff scheduling, which reduced the software build needs. Prebuilt provider calendar templates with well-child visits were being used by children’s providers at the Mayo Clinic long before self-scheduling was implemented. Thus, self-scheduling of this visit type required very little change management other than communication of the change. Self-schedulers were able to see an open well-child visit they wanted and could book it. A subsequent informal provider survey confirmed that most providers (20/25, 80%) were unaware that a proxy had booked the appointment.

Well-child appointments also did not need a provider order to initiate scheduling. Many primary care visits involve lab and radiology procedures, which require orders for scheduling. This is needed to identify providers able to *close the loop* on imaging and lab test results. Preventive services such as screening mammography and chronic disease visits for diabetes, hypercholesterolemia, and hypertension are examples of visits requiring orders. Screening mammograms require radiology visit orders, and laboratory orders (eg, hemoglobin A_1c_, lipids, etc) are often requested in advance of chronic disease appointments. Chronic disease visits also have some appointment length challenges because many patients have multiple chronic diseases, so appointment lengths often need to be individualized. The well-child visit was a good candidate for self-scheduling; it required no decision support for appointment urgency or appointment duration, and it did not require an order before scheduling.

### Scheduling Rules in Software

The scheduling rules in the software for the well-child visit include the following:

1. Frequency limitations of appointments: the software looks back at the date of previous well-child appointments to ensure that frequency limitations are met.
2. Age: limited to ages 2-12 years.
3. Assigned primary care provider: children need an assigned primary care provider to be eligible. To ensure continuity of care, there is no option to schedule a well-child appointment with any provider other than the assigned primary care provider. The software automatically pulls the primary care provider scheduling template.
4. Appointment lead time: calendar availability was 12 weeks in the future. Provider templates were built for no more than 12 weeks in the future for the Rochester, Minnesota, site; therefore, specific appointment times beyond 12 weeks could not be self-scheduled at that site. Although the Northwest Wisconsin site had provider calendar templates available for more than 12 weeks, the self-scheduling rules for the initial implementation did not account for the expanded scheduling ability at the Northwest Wisconsin location.

### Appointment Definitions and Data

Individual appointment actions are dichotomously classified as a *schedule* or *cancel* action. A schedule action reserves a single appointment time; a cancel action opens a previously scheduled appointment time. Well-child visits are typically 30 minutes but could be scheduled for 45 minutes by staff schedulers.

Staff schedulers are clinic staff employees who schedule or cancel appointments for patients. Self-schedulers or self-cancellers were the parent or proxy who used the Mayo software interface (web or mobile) to self-schedule or self-cancel the child’s appointments. It should be noted that we focused on self-scheduled actions in this paper. Some proxies did not use the self-scheduling feature but self-cancelled appointments made by staff schedulers. To be considered self-scheduled, a patient had to have at least one appointment action of self-scheduling (booking an appointment with the self-schedule software). The few patients who self-cancelled their staff-scheduled appointments were classified as staff scheduled.

**Figure 1:**
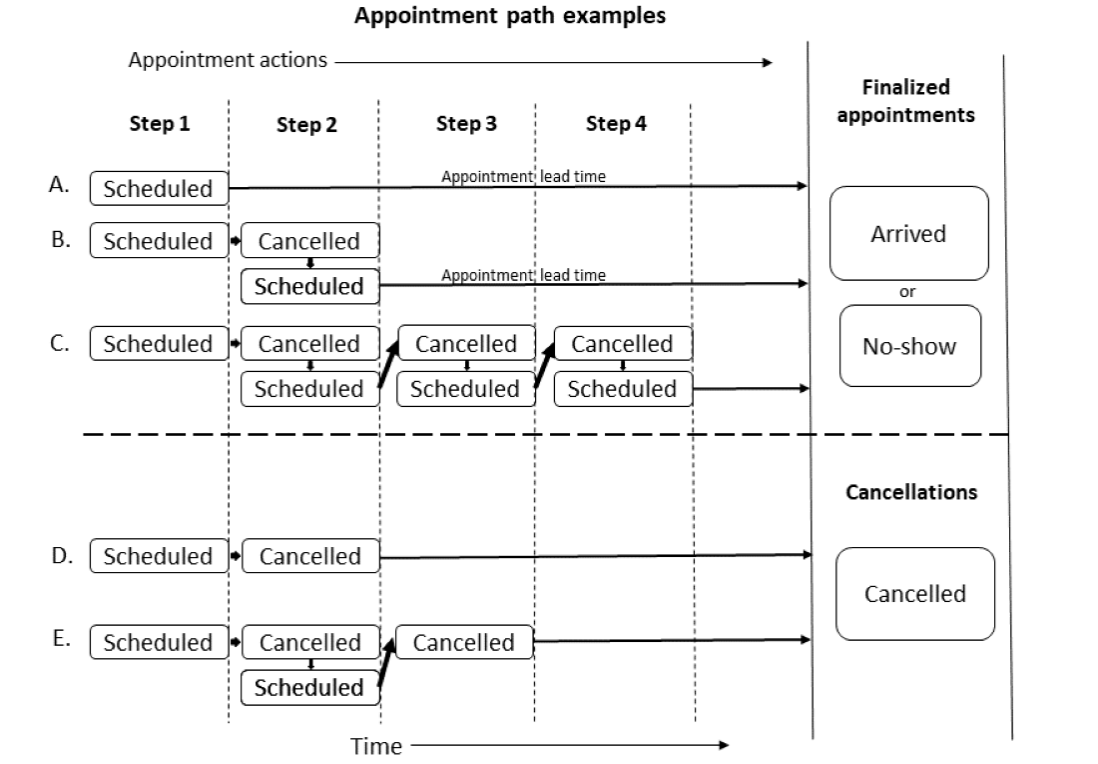
Examples of different appointment paths showing the appointment actions and appointment steps leading to a finalized appointment or cancellation.

An appointment path is the sequence of appointment actions leading to a finalized appointment or cancellation outcome. Finalized appointments were those scheduled appointments that were left scheduled up to the appointment date and time (not cancelled before the appointment time). Figure 1 shows examples of appointment paths and appointment outcomes. Our data start with a time-stamped appointment schedule action. We dichotomized appointment actions into those created by staff schedulers and those created by self-scheduling. As shown in Figure 1, each patient (whether self-scheduled or staff scheduled) begins with a scheduling action that we term appointment step 1. Patients can then go through several decision steps of whether to cancel or reschedule (a cancel and schedule pair). Some patients would cancel and reschedule multiple times before a finalized appointment. To quantify this activity, we counted the appointment steps, as shown in Figure 1. Example (A) within Figure 1 shows an appointment path to appointment finalization with just 1 step, the initial scheduling action. Examples (B) and (C) within Figure 1 show appointment paths for appointment finalization taking 2 and 4 steps, respectively. Appointment paths ending in a cancellation outcome may also take several appointment steps. Figure 1 shows examples (D) and (E) that take 2 and 3 appointment steps, respectively, to a cancellation.

Appointment outcomes are dichotomously categorized as finalized appointments or cancellations. Finalized appointments are further dichotomously categorized as completed or no-show (never arrived at the scheduled appointment time). The well-child visit appointment was an in-person visit; therefore, this study did not include any telephonic or video appointments.

Figure 1 example (A) also shows the appointment lead time, which is the scheduled appointment date and time minus the date and time the appointment was made. This is the lead time that the patient has from the date of scheduling the appointment to the actual future reserved appointment date.

### Data Collection and Study Metrics

We used appointment data sets from the Mayo Clinic Enterprise Office of Access Management in this study. The data set captured all appointment activity dichotomously as a scheduling or cancellation action, and whether the scheduling or cancellation action was done by the appointment staff or self. We obtained complete scheduling and cancellation actions for all well-child visits encompassing ages 2 to 12 years from February 1, 2019, through February 28, 2020. The time of the appointment action (scheduling or cancelling) was included in the data set and was categorized as weekend (Saturday or Sunday) and after-hours weekday (not occurring within 7 AM to 5 PM). There were mobile and web versions for self-scheduling, and we were able to capture which was used for each self-scheduled action. If a patient used the web version on a mobile device, it was captured as web use.

Demographic information was obtained from all children whose proxy or proxies either cancelled or made a well-child appointment for the 13 months of the study. Demographic data on the proxies were not collected for this study.

Finalized appointment outcomes were obtained from a final data set that contained only scheduled appointments still in the system on the day of the expected visits. Appointments cancelled any time up to the appointment date and hour were excluded from the finalized appointment outcome analysis to leave behind only those scheduled visits that providers expected to see face-to-face. The finalized appointment outcomes were dichotomously categorized as no-show or arrived.

### Statistics and Ethics

We used JMP Pro 14.2 (SAS) for descriptive statistics and statistical analyses. For comparison between categorical variables, we used chi-square tests and odds ratios (ORs). This study met the criteria for institutional review board exemption (20-006809).

## Results

### Well-Child Visit Scheduling Counts and Provider Counts

There were 36,392 well-child scheduling actions for 399 providers. Pediatric providers accounted for 65.20% (23,727/36,392) and family medicine providers for 34.80% (12,665/36,392) of the well-child scheduled visits. We limited this study to those who could access self-scheduling, so only those patients with proxy access to POS (portal registration) were included. This resulted in 24,417 scheduling actions for analysis, with 4.92% (1201/24,417) of all scheduling actions being self-scheduled.

### Patient Characteristics

**Figure 2:**
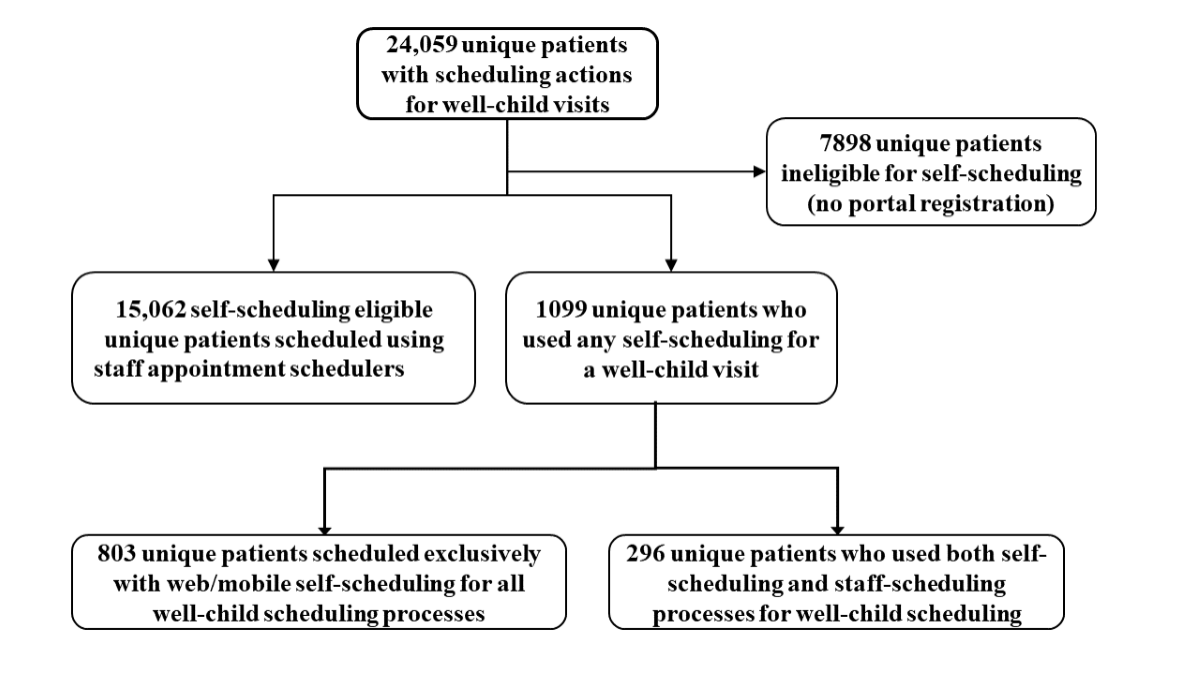
Patient counts by category of those who completed well-child visit scheduling actions during the 13 month study period.

Figure 2 shows the unique patient counts of those who scheduled well-child appointments during the study. There were 32.83% (7898/24,059) of patients who could not self-schedule because they did not have POS registration. Of the 16,161 patients who had portal access, 6.80% (1099/16,161) used self-scheduling. Of the 1099 patients who used self-scheduling, 73.1% (803/1099) used self-scheduling and self-cancelling exclusively, and 26.9% (296/1099) had appointment actions that used both self-scheduling and staff scheduling.

Sex, race, and ethnicity of children were not statistically different between those who were self-scheduled and those who were not (Table 1). However, self-scheduled appointments were proportionately greater for those aged 6 to 12 years than staff-scheduled appointments (Table 1).

**Table 1:** Demographic comparison of patients with portal registration with well-child appointment actions (N=16,161). The self-scheduled group completed at least one self-scheduling action. Demographics compared are only those with access to self-scheduling (those without portal registration were not included).

Demographics		Self-scheduled (n=1099), n (%)	Staff scheduled (n=15,062), n (%)	*P* value^a^
**Age (years)**				<.001
	2-5	480 (43.68)	8523(56.59)	
	6-12	619 (56.32)	6539 (43.41)	
Female sex		524 (47.68)	7290 (48.40)	.64
**Race**				.27
	White	963 (87.63)	12,987 (86.22)	
	Black	20 (1.82)	374 (2.48)	
	Asian	31 (2.82)	545 (3.62)	
	Other or not disclosed	85 (7.73)	1156 (7.67)	
**Ethnicity**				.97
	Hispanic	41 (3.73)	585 (3.88)	
	Not Hispanic	1026 (93.36)	14,041 (93.22)	
	Undisclosed or unknown	32 (2.91)	436 (2.89)	

^a^Null hypothesis (H^0^): patient demographic proportions are equal.

### After-Hours Scheduling and Appointment Lead Time

The 1099 patients who used the self-scheduling feature generated 1490 appointment actions (1201 self-scheduling actions and 289 cancelling actions), resulting in 912 finalized appointments. Similarly, 15,062 patients who used staff scheduling generated 29,604 appointment actions (23,216 scheduling actions and 6388 cancelling actions), resulting in 16,828 finalized appointments. Cancelling actions in the self-scheduled group accounted for 19.4% (289/1490) of all appointment actions in that group and 21.58% (6388/29,604) in the staff-scheduled group (*P*=.046). The differences in scheduling between self-scheduled and staff-scheduled actions are shown in Table 2. There were across-the-board differences when scheduling actions occurred on weekend days and weekdays after usual staff scheduler hours and when scheduling lead time was greater than 12 weeks. As noted in the *Methods* section, staff scheduler hours were mostly limited to usual outpatient weekday hours, so staff-scheduling actions on weekend days and after hours on weekdays were expected to be low; 12.99% (3015/23,216) of staff-scheduling actions had appointment lead times greater than 12 weeks. As noted in the *Methods* section, a software rule excluded self-scheduling with lead times over 12 weeks; thus, patients wanting a longer lead time had to be scheduled by staff.

**Table 2:** Comparison of self- versus staff-scheduling actions (does not include cancelling actions). Scheduling actions are limited to those who could access self-scheduling (those with portal registration).

Appointment metric	Self-scheduled (scheduling actions only), n (%)	Staff scheduled (scheduling actions only), n (%)	*P* value^a^
Scheduling appointment action count	1201 (100)	23,216 (100)	N/A^b^
Any appointment-scheduling action outside regular business hours (Monday-Friday, 7 AM to 5 PM)	354 (29.48)	199 (0.86)	<.001
Appointment-scheduling action on weekdays (Monday-Friday) outside of 7 AM to 5 PM	227 (18.90)	180 (0.78)	<.001
Appointment-scheduling action on weekend days (Saturday or Sunday)	127 (10.57)	19 (0.08)	<.001
Scheduling action lead time over 12 weeks	0 (0)	3015 (12.99)	<.001

^a^Null hypothesis (H^0^): proportions are equal between self-scheduled and staff scheduled.

^b^N/A: not applicable.

### Staff Scheduler Work Involved in Self-Scheduled Appointments

As indicated in Figure 1, appointment scheduling can go through many steps of scheduling and cancelling over the span of a finalized or cancelled appointment. A patient could use both self-scheduling and self-cancelling for parts of the appointment steps and staff scheduling and staff cancelling for other parts of the appointment steps, leading to a single finalized appointment. Self-scheduling would be inefficient if patients who self-scheduled also relied on staff schedulers to cancel or reschedule the appointment. To determine whether staff were involved in the rework of this type, we examined all finalized appointments to determine how much of the scheduling and cancelling work was being done by staff and how much was being done by the patients themselves. Table 3 shows that of the 912 finalized appointments with any self-scheduling activity, only 9.9% (147/1490) involved a staff scheduler. Thus, self-scheduling activity did not lead to large amounts of rework by staff schedulers to obtain a finalized appointment. Table 3 also shows that there were on average 1.63 appointment actions per finalized appointment for those with self-scheduling activity, with only 0.16 actions per finalized appointment attributable to staff schedulers. In contrast, on average, staff schedulers took 1.76 appointment actions for each staff-scheduled finalized appointment.

**Table 3:** Comparison of the average patient-performed appointment actions per finalized appointment for self-scheduled and staff scheduled.

Appointment metric	Self-scheduled (but staff could cancel)	Staff scheduled (but the patient could cancel on the web)
Total appointment actions (schedule and cancel), n	1490	29,604
Scheduling actions, n (%)	1201 (80.6)	23,216 (78.4)
Cancelling actions, n (%)	289 (19.4)	6388 (21.6)
Appointment actions (schedule or cancel action) performed by patient or proxy (web or mobile), n (%)	1343 (90.1)	540^a^ (1.8)
Appointment actions performed by Mayo scheduler, n (%)	147^b^ (9.9)	29,064 (98.2)
Appointments finalized, n (remaining on calendar up to visit date and time)	912	16,828
Average patient or proxy performed appointment actions per finalized appointment (total count of patient or proxy appointment actions divided by total count of finalized appointments)	1.47 (1343/912)	0.032 (540/16,828)
Average Mayo scheduler actions per finalized appointment (total count of Mayo scheduler actions divided by total count of finalized appointments)	0.16 (147/912)	1.73 (29,064/16,828)
Average appointment actions per finalized appointment (total count of appointment actions divided by total count of finalized appointments)	1.63 (1490/912)	1.76 (29,604/16,828)

^a^Staff scheduled but was self-cancelled.

^b^Self-scheduled but had cancel actions taken by staff schedulers (however, 142 of the 289 cancels were self-cancelled).

### Comparison of Appointment Steps With Finalized Appointment

**Figure 3:**
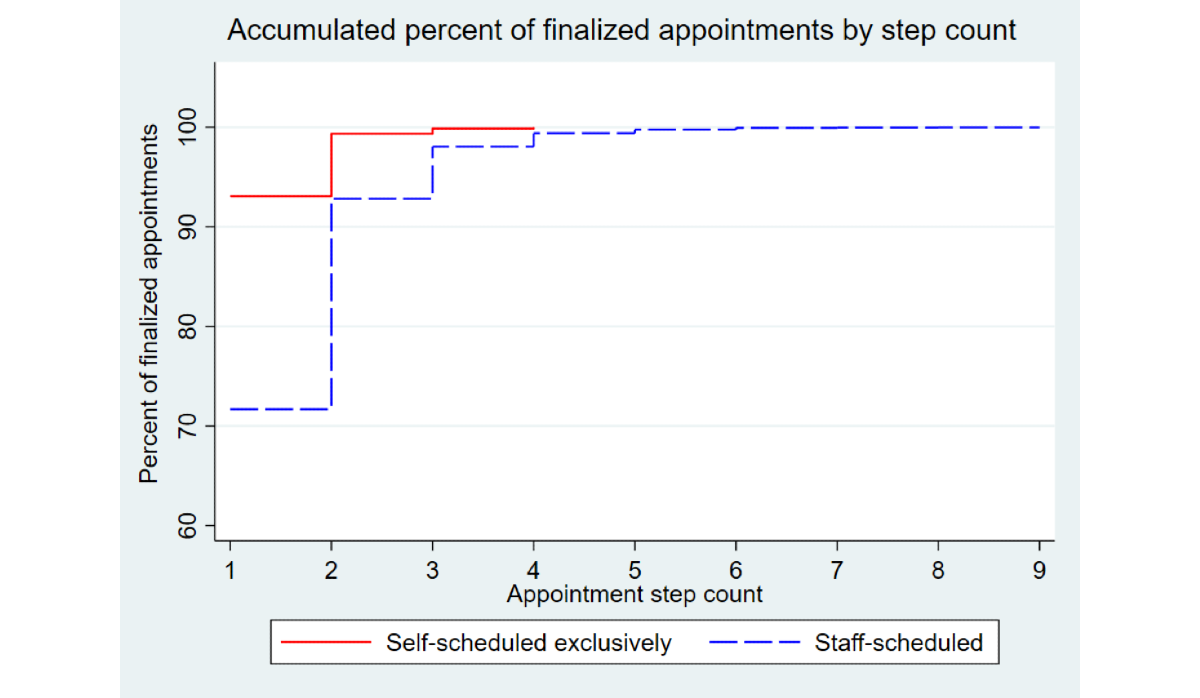
Accumulated percent of finalized appointments by step count.

As shown in Figure 1, the initial appointment step starts with scheduling a future appointment, which we term appointment step 1. In addition, as indicated in Figure 1, there may be multiple steps before a finalized appointment. Figure 3 shows a comparison between exclusively self-scheduled and staff-scheduled appointments on the accumulated appointment steps taken before appointments were finalized. To make our analysis comparable, we limited it to only those patients who had a single finalized appointment within the timeframe of the study. A total of 765 patients completed a single appointment exclusively using self-scheduled appointment actions. Of those, 93.07% (712/765) finalized the appointment in a single step; for the 13,168 patients who used staff schedulers for a single appointment, 71.68% (9439/13,168) finalized the appointment in a single step (*P*<.001). Thus, 28.32% (3729/13,168) of the staff-scheduled appointments required multiple appointment steps compared with 6.93% (53/765) requiring multiple steps for a self-scheduled appointment (OR 5.3, 95% CI 4.0-7.0).

### Comparison of Finalized Appointment Outcomes

Of the 1201 self-scheduling appointment actions, 912 became finalized appointments. Self-scheduling accounted for 5.14% (912/17,740) of finalized, well-child appointments. Table 4 shows the differences in appointment outcomes for those who had finalized appointments. No-shows for well-child appointments were not statistically different between self-scheduled and staff-scheduled appointments. As with scheduling actions, we found a significant number of staff-scheduled finalized appointments (1876/16,828, 11.15%) involved appointment lead times over 12 weeks.

**Table 4:** Comparison of appointment outcomes for finalized appointments (those remaining scheduled on the day of appointment).

Visit outcome	Self-scheduled	Staff scheduled	*P* value^a^
Finalized appointments (scheduled and not cancelled before the visit date), n (%)	912 (100)	16,828 (100)	N/A^b^
Arrived for appointment (percent of patients seen for visit day appointment), n (%)	884 (96.93)	16,135 (95.88)	.12
No-show (percent not arriving at the appointment), n (%)	28 (3.07)	693 (4.12)	.12
Appointment lead time greater than 12 weeks (84 days), n (%)	0 (0)	1876 (11.15)	<.001
Median appointment lead time (days)	27	32	N/A

^a^Null hypothesis (H^0^): proportions are equal between self-scheduled and staff-scheduled appointments.

^b^N/A: not applicable.

### Mobile-Based Versus Web-Based Self-Scheduling

For the 1201 self-scheduled appointment actions, 61.20% (735/1201) were completed through the patient web application and 38.80% (466/1201) were completed through the mobile app. Of the 912 appointments finalized, 60.2% (549/912) were through the web and 39.8% (363/912) were through the mobile app.

## Discussion

### Principal Findings

For those with the opportunity to self-schedule (registered with POS), 5.14% (912/17,740) of finalized well-child visits were self-scheduled. For those with portal registration, no-show rates were statistically similar to those who did not self-schedule. Self-scheduling occurred 29.5% (354/1201) of the time outside of the usual business hours. There was a significant demand for appointment lead times greater than the 12-week window allowed in self-scheduling, demonstrated by 11.15% (1876/16,828) of staff-scheduled appointments with lead times over 12 weeks. Self-scheduling resulted in a significantly higher percentage of single-step appointment scheduling (*one and done*) than with staff scheduling.

### Practice Implications

Although it was outside the scope of this study to examine cost, a significant number of patients (n=803) exclusively used self-scheduling, likely decreasing scheduling expenses for that group. For patients using any self-scheduling, there were only 9.87% (147/1490) of scheduling actions performed by staff schedulers, so there was little indication of unintended consequences leading to more staff work.

Paré et al [10] found that the flexibility of being able to book appointments when it was most convenient was one of the highest patient-perceived benefits of the e-booking system they studied. Ballantyne et al [11] also noted that parents of special needs children thought it was important to be able to self-schedule appointments with a computer or mobile device. With 29.5% (354/1201) of our self-scheduling occurring outside of normal business hours and 38.8% (466/1201) of self-scheduling by mobile devices, many parents or proxies were able to access and complete scheduling activities anytime and anywhere. With this increase in scheduling convenience, it is possible that self-scheduling can significantly improve patient satisfaction.

With the amount of self-scheduling that occurred after hours, our findings might help decide whether to have appointment schedulers on duty during evenings and on weekend days. This could be helpful for scheduling patients who do not have portal access and those who have access to self-schedule but may need additional help.

### No-Shows Associated With Self-Scheduling

Although no-shows were less frequent in the self-scheduled group, this was not statistically significant. In other studies, self-scheduling has been associated with lower no-show rates [10,12-14]. Our finding that missed appointments in self-scheduled patients were not statistically less suggests that self-scheduling itself may not decrease missed appointments. It should be noted that at the Mayo Clinic, patients receive appointment reminders by text or phone for all appointments, whether self-scheduled or staff scheduled.

### Appointment Lead Time

The median appointment lead times were about 1 month in both the self-scheduled and staff-scheduled groups (Table 4). Self-scheduled lead times greater than the 12-weeks were not allowed by the software. However, it is notable that 11.15% (1876/16,828) of staff-scheduled finalized appointments had an appointment lead time greater than 12 weeks. For the subgroup of Northwest Wisconsin, where appointment lead times up to 6 months were available using staff scheduling, 27.25% (1759/6455) had a lead time of over 12 weeks. This suggests that there might be increased uptake in self-scheduling if future software updates can accommodate longer lead times.

### Comparison With Previous Studies

Our 5% uptake is similar to that observed by Zhang et al [15], who found an uptake of 4% after 29 months of using an e-appointment–scheduling system in an Australian primary health care clinic. In that study, they found that many patients did not see the value of the e-appointment system when they could easily call by phone to make appointments, and the patients noted limitations in the functionality of the self-scheduling system [15].

Lack of awareness of the self-scheduling feature has been an issue with implementation elsewhere. Cao et al [16] noted that 53% of patients were unaware of their web-based appointment system. Although we attempted to increase patient awareness of the ability to self-schedule this visit type, we do not know how many who needed appointments were actually aware of the self-scheduling option.

### Lessons for Future Enhancements

As a large percentage of the staff-scheduled visits were made with more than a 12-week lead time, there is likely a significant demand for this to be incorporated into the self-scheduling of this visit type. Software enhancement could be made to set future visit requests greater than 12 weeks in a placeholder visit and then automatically generate a reminder to the patient as soon as appointment templates are opened for scheduling. For our Mayo Clinic Northwest Wisconsin site, where provider schedule templates are built out 6 months in advance, there may be a trade-off in provider satisfaction for those who do not want to be locked into a 6-month schedule. Another option could be to have a pool of providers available to give more scheduling options, but for the well-child visit, this may negate the advantage of continuity of care [17-19].

### Limitations

Our study was limited to just 1 self-scheduled appointment, the well-child visit, limiting the potential generalizability of our results. There are numerous other types of visits, which may have different results. The study also took place in a majority White community, so there may be differences in communities with different demographics. To control for portal access, we included only those with portal registration; there would be a smaller percentage engaged in self-scheduling had we included those without access to self-scheduling. The scheduling platform we used (Mayo Clinic POS) is specific to the Mayo Clinic, but appointments and rules were managed with information from Epic, the Mayo Clinic’s EHR, which has a wide user base across the United States.

The uptake of self-scheduling may also differ in other practices. Although there was some promotion of this new module, additional promotion may have resulted in a larger uptake. It is possible that the uptake of self-scheduling was influenced by the advantage of 24/7 availability compared to the more limited availability of Mayo Clinic schedulers (mostly 7 AM-5 PM on weekdays). Self-scheduling uptake could be lower if staff schedulers were available during the evening hours and on weekends when some of the self-scheduling occurred.

### Future Research Implications

The impact of self-scheduling on patient satisfaction is unclear. At the Mayo Clinic, patient satisfaction with access significantly decreased, for a time, associated with an EHR switch [20]. It is possible that the ability to self-schedule could also be associated with a measurable change in patient satisfaction. Future research is also needed on patient acceptance of self-scheduling, especially in view of some studies that have shown patients’ reluctance to use a self-scheduling feature [3,15,21].

Our study showed that the impact of self-scheduling on no-show rates was not significant when limited to those with portal registration. In a systematic review by Dantas et al [22], longer appointment lead times were found to be a major driving factor for higher no-show rates. It deserves restatement that the well-child visit is a special visit type where, as we show in this study, proxies may find a long lead time desirable. Additional research is needed to clarify the confounders related to self-scheduling and no-show rates. Additional investigation is also needed for other types of self-scheduled appointments (such as acute care visits) for more generalizable conclusions on self-scheduling quality and safety issues.

### Conclusions

Well-child appointments were successfully scheduled entirely within the appointment software, resulting in fewer interactions with appointment schedulers, frequently outside of the hours staff schedulers usually work. Self-scheduled appointments were more likely to be completed in a single appointment step than staff-scheduled appointments; self-schedulers were unlikely to need additional help from a scheduler to finalize an appointment. Self-scheduled appointment no-show rates were not statistically different from those of staff-scheduled appointments. Self-scheduling software may need to accommodate patients wanting to schedule appointments further in the future than their providers’ appointment templates allow.

## Abbreviations

EHR: electronic health record
OR: odds ratio
POS: Patient Online Services
